# Overview of non‐coding mutations in chronic lymphocytic leukemia

**DOI:** 10.1002/1878-0261.12416

**Published:** 2019-01-04

**Authors:** Valeria Spina, Davide Rossi

**Affiliations:** ^1^ Laboratory of Experimental Hematology Institute of Oncology Research Bellinzona Switzerland; ^2^ Division of Hematology Oncology Institute of Southern Switzerland Bellinzona Switzerland

**Keywords:** chronic lymphocytic leukemia, mutational analysis, non‐coding region

## Abstract

Chronic lymphocytic leukemia (CLL) is the most frequent leukemia type in which the genetic alterations influencing the clinico‐biological course are not entirely understood. CLL has a heterogeneous course, with some patients showing an indolent course and others experiencing an aggressive course. Whole‐genome sequencing and whole‐exome sequencing studies identified recurrently mutated genes in CLL and profiled its clonal evolution patterns. However, more recent whole‐genome sequencing studies also identified variants in non‐coding sequences of the CLL genome, revealing important lesions outside the protein‐coding regions. Here we describe the most representative non‐coding lesion of the CLL genome, including lesions in the 3′‐UTR region of *NOTCH1* which result in the truncation of the NOTCH1 protein PEST domain, and non‐coding mutations in an enhancer region on chromosome 9p13 which result in reduced expression of the PAX5 transcription factor. In addition, we describe the role of microRNA in CLL, in particular the miR15a/miR16‐1 microRNA recurrently affected by deletions of chromosome 13q14. Together, new findings in non‐coding genome genetic lesions provide a more complete portrait of the genomic landscape of CLL with clinical implications.

**Abbreviations**
AID: activation‐induced deaminase
BCR: B‐cell receptor
BCL2: B‐cell lymphoma 2
CLL: chronic lymphocytic leukemia
DLBCL: diffuse large B‐cell lymphoma
FL: follicular lymphoma
IGHV: immunoglobulin heavy variable
IL: interleukin
MCL: mantle cell lymphoma
M‐CLL: mutated chronic lymphocytic leukemia
NF‐κB: nuclear factor‐κB
NGS: next generation sequencing
PFS: progression‐free survival
SHM: somatic hypermutation
U‐CLL: unmutated chronic lymphocytic leukemia
WES: whole‐exome sequencing
WGS: whole‐genome sequencing

## 1 Introduction

Chronic lymphocytic leukemia (CLL) is a common B‐cell tumor of adults; at diagnosis, the median age of patients is 72 years. Patients have been classically categorized depending on B‐cell receptor (BCR) immunoglobulin expression: the immunoglobulin heavy‐chain variable region gene (IGHV) harboring somatic hypermutation (SHM; IGHV‐mutated) group and the immunoglobulin heavy‐chain variable region gene harboring unmutated (IGHV‐unmutated) group (Damle *et al*., 1999; Hamblin *et al*., 1999). Patients with tumor clones with < 2% difference from germline or no mutation in the IGHV‐unmutated gene have a poorer prognosis compared with patients with the IGHV‐mutated gene (Damle *et al*., 1999; Hamblin *et al*., 1999).

Deletion of chromosome 13q14, del(13q14), is the most frequent cytogenetic aberration in CLL, occurring in ~ 55% of newly presented cases; if occurring as the sole genetic abnormality, it is associated with a benign course. Deletion of chromosome 11q, del(11q), occurs in ~ 25% of progressive but previously untreated patients, and in ~ 10% of early‐stage patients (Quesada *et al*., 2011; Zenz *et al*., 2010). Deletion 11q targets the *ATM* gene, which encodes for the proximal DNA damage response kinase ATM. Trisomy 12 occurs in ~ 15% of newly presented cases (Seiffert *et al*., 2012). Deletion of chromosome 17p, del(17p), occurs in ~ 7% of newly presented cases. Deletion of chromosome 17p targets the *TP53* gene and is associated with chemoresistance (Hallek *et al*., 2010). Genomic studies have disclosed the complexity of cancer clonal architecture and identified several genetic prognostic biomarkers that are significantly associated with CLL overall survival, time to first treatment in cases managed with watch‐and‐wait, or progression‐free survival (PFS) in treated cases (Crespo *et al*., 2003; Damle *et al*., 1999; Döhner *et al*., 2000; Malek, 2013).

Whole genome/exome sequencing (WES/WGS) studies in CLL revealed recurrently mutated driver genes such as *NOTCH1*,* MYD88*,* TP53*,* ATM*,* SF3B1*,* FBXW7*,* POT1*,* CHD2*,* RPS15*,* IKZF3*,* ZNF292*,* ZMYM3*,* ARID1A* and *PTPN11* (Fabbri *et al*., 2011; Landau *et al*., 2015; Puente *et al*., 2011, 2015; Quesada *et al*., 2011; Ramsay *et al*., 2013; Rossi *et al*., 2012). Important benefits of WGS studies included the identification of variants in non‐coding sequences of the CLL genome, revealing important lesions outside the protein‐coding regions which could help to disclose, together with the coding genetic lesion, the complexity of the CLL genetic landscape.

## 2 The miR‐15a and miR16‐1 in CLL

**Figure 1:**
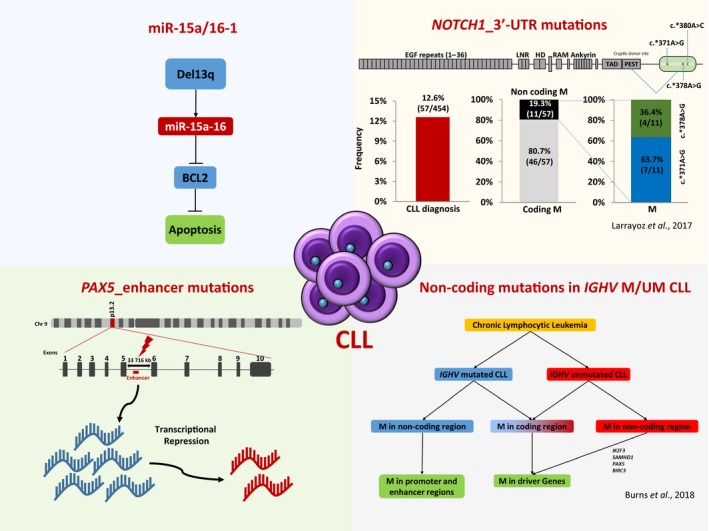
Non‐coding mutations in CLL. Portrayal of the most representative non‐coding lesion of the CLL genome.

The identification of the epicenter of the minimal deleted region loss, ~ 30 kb on chromosome 13q, revealed the first example of non‐coding region alteration in CLL. The del13q14 leads to the monoallelic loss of the microRNA, miR15a and miR16‐1 (Fig. 1) (Calin *et al*., 2002). In normal cells, miR15a and miR16‐1 downmodulate at the post‐transcriptional level the expression of key regulators of apoptosis and cell cycle. The notion that miR15a/16‐1 and BCL2 expression levels are inversely correlated in CLL, and that downregulation of miR15a/16‐1 results in an increase of B‐cell lymphoma 2 (BCL2) expression, with consequent inhibition of apoptosis, led to the identification of BCL2 as the primary target of miR15a/16‐1 (Table 1) (Cimmino *et al*., 2005; Cory and Adams 2002, 2005; Sanchez‐Beato *et al*., 2003). Consistently, the miR15a/16‐1 consensus regions on the BCL2 mRNA disclosed that these two microRNA species are direct negative regulators of BCL2 at the post‐transcriptional level (Fig. 1) (Calin *et al*., 2008). The miR15a/16‐1 conditional deletion in mouse B‐cells results in the development of a CLL‐like monoclonal CD5^+^ lymphocyte proliferation in 40% of mice, proof of its involvement in CLL pathogenesis (Klein *et al*., 2010).

**Table 1:** Summary of non‐coding lesions in CLL

Reference	Genomic region	Genes	Mutations	Functional consequences	Pathways
Cimmino *et al*. (2005)	del13q14	*miR‐15a miR16‐1*	–	miR‐15a/16‐1 downregulation in leukemic cell lines resulted in an increase of BCL2 expression with consequent inhibition of apoptosis	Cell cycle
Klein *et al*. (2010)	del13q14	*miR‐15a miR16‐1*	–	miR‐15a/16‐1 deletion in mice developed a CLL‐like monoclonal CD5+ lymphocyte proliferation	Cell cycle
Puente *et al*. (2015)	3′‐UTR	*NOTCH1*	c.*371A>G	This mutation is predicted to remove a PEST domain of NOTCH1 and to increase protein stability	NOTCH
Puente *et al*. (2015)	9p13	*PAX5*	–	Decrease in PAX5 expression	BCR

## 3 *NOTCH1* coding and non‐coding mutations

*NOTCH1* encodes a class I transmembrane protein which acts as a ligand‐activated transcription factor that plays a key role in cell proliferation, differentiation and apoptosis (Paganin and Ferrando, 2011). NOTCH1 binds to its ligand and then undergoes proteolytic cleavages which enable its intracellular domain to translocate into the nucleus to mediate transcriptional activation of multiple target genes, including *TP53*,* MYC* and genes which encode components of the nuclear factor κB (NF‐κB) pathway. Most CLL mutations which affect *NOTCH1* are frameshift or nonsense events which are clustered within exon 34 (including a highly recurrent c.7544_7545delCT deletion) and are selected to disrupt the PEST domain of the protein (Fabbri *et al*., 2011; Rossi *et al*., 2012; Sportoletti *et al*., 2010). As the PEST domain is required to limit both the intensity and duration of NOTCH1 signaling activation, its removal is predicted to impair NOTCH1 degradation, thereby resulting in the accumulation of an active NOTCH1 isoform and subsequent deregulation of signaling (Fabbri *et al*., 2011, 2017; Sportoletti *et al*., 2010). Consistent with this prediction, multiple cellular pathways, including those controlling cell metabolism and cell cycle progression, are deregulated in CLL harboring *NOTCH1* mutations (Del Giudice *et al*., 2012; Fabbri *et al*., 2017; Puente *et al*., 2011). NOTCH1 is preferentially targeted in specific clinical and biological groups of CLL (Sportoletti *et al*., 2010), including cases that have developed into Richter syndrome (Rossi *et al*., 2012; Villamor *et al*., 2013), cases harboring unmutated‐IGHV genes, cases harboring a subset eight BCR configuration, and cases harboring trisomy of chromosome 12 (Del Giudice *et al*., 2012; Fabbri *et al*., 2011; Rossi *et al*., 2012, 2013; Sportoletti *et al*., 2010).

Among CLL harboring *NOTCH1* mutations, treatment with FCR, R‐Clb or O‐Clb does not result in the expected increase in PFS compared with treatment with FC or with chlorambucil alone (Stilgenbauer *et al*., 2014). These observations point to *NOTCH1* mutations as a biomarker of resistance to the anti‐CD20 antibodies rituximab and ofatumumab in CLL. The outcome of CLL patients treated with obinutuzumab combined with chlorambucil improves independently of *NOTCH1* mutation status, suggesting that the augmented cytotoxicity of obinutuzumab or the increased dose of the anti‐(CD20) IgG1 antibody used in the obinutuzumab‐chlorambucil schema overcomes *NOTCH1* mutation‐associated resistance to rituximab (Estenfelder *et al*., 2016). The mechanism underlying the anti‐CD20 refractoriness associated with *NOTCH1* mutations remains obscure.

NOTCH1 is broadly activated in CLL, where most of the cases express the intracellular active portion of the NOTCH1 protein, despite the absence of coding gene mutations. A proportion of cases lacking coding gene mutations but having biochemical clues of NOTCH1 activation is now justified by the occurrence of 3′‐UTR mutations in the non‐coding region of the *NOTCH1* exon 34 (Fabbri *et al*., 2017). Non‐coding mutations in the 3′‐UTR of *NOTCH1* (c.*371A>G) have been also described in 2–4% of cases of CLL (Fig. 1) (Bittolo *et al*., 2017; Nadeu *et al*., 2016; Puente *et al*., 2015). The 3′‐UTR of *NOTCH1* mutation leads to a novel splicing event between a cryptic donor site located in the coding region of *NOTCH1* exon 34 and a newly created acceptor site in the 3′‐UTR, resulting in a deletion that includes the last 158 coding bases. This within‐exon splicing is predicted to remove a PEST domain of *NOTCH1* and to increase protein stability, as previously described for *NOTCH1* mutation affecting exon 34 (Table 1) (Rossi *et al*., 2012). The 3′‐UTR mutations are mutually exclusive of other *NOTCH1* somatic variants, consistent with the notion that they are selected by the tumor as an alternative genetic mechanism of the PEST domain deletion. Indeed, CLL cells having the 3′‐UTR mutations or the exon 34 coding mutations display constitutive levels of cleaved and active NOTCH1 protein (D'Agaro *et al*., 2017). Besides mimicking the biological effect of exon 34 mutations, the 3′‐UTR non‐coding mutation of *NOTCH1* also has the same clinical consequences (Puente *et al*., 2015).

### 3.1 Clinical impact of *NOTCH1* non‐coding mutations

*NOTCH1* coding mutations identified in CLL affect exon 34 and include the highly recurrent c.7544_7545delCT deletion (Arruga *et al*., 2014; Puente *et al*., 2011; Rosati *et al*., 2009). At diagnosis, these mutations occur in ~ 8% of cases and have a high prevalence in advanced disease stages, in treatment‐refractory disease and after transformation to Richter syndrome (Baliakas *et al*., 2015; Oscier *et al*., 2013). The prognostic value of *NOTCH1* non‐coding mutations was validated in chemotherapy first‐line treatment patients by the UK CLL4 trial study (Larrayoz *et al*., 2017) (Fig. 1). That study also showed that *NOTCH1* non‐coding mutations together with coding mutation increase the power to predict outcomes in CLL patients. Taken together, these studies support analysis of *NOTCH1* non‐coding region in order to stratify reduced survival patients better and to identify patients predestined to respond poorly to rituximab treatments (Stilgenbauer *et al*., 2014).

## 4 Non‐coding mutations affecting *PAX5* gene

The B‐cell specific activator protein, also known as PAX5, is a transcription factor and is an important B‐cell precursor for normal B‐cell differentiation and maturation (Nutt *et al*., 2001). *PAX5* gene expression is involved in IGHV gene rearrangement, BCR signal transduction and B‐cell survival, so deletion or inactivation of *PAX5* gene led to cell arrest in Pro‐B‐cell stage. PAX5 heterozygous mice showed an accumulation of interleukin (IL)7‐dependent proB cells and developed B‐ALL when endangered by infections (Martin‐Lorenzo *et al*., 2015). Furthermore, *PAX5* translocations and mutations have been observed in B‐cell lymphomas and B‐ALL (Mullighan *et al*., 2007; Poppe *et al*., 2005).

*PAX5* recurrent mutations in enhancer non‐coding regions have been recently associated to activity alterations of the gene (Fig. 1) (Puente *et al*., 2015). A large WGS study of 150 CLL patients identified non‐coding mutations in the *PAX5* enhancer region on chromosome 9p13. Patients harboring non‐coding mutations in this region showed a pronounced decrease in *PAX5* expression compared with *PAX5* wild‐type patients (Table 1) (Puente *et al*., 2015). By using the CRISPR/Cas9 approach to target the *PAX5* enhancer region in an indicative cell line, they also showed a decrease in *PAX5* expression. The study identified 42/506 (8%) CLL‐mutated samples in other B‐cell lymphomas such as diffuse large B‐cell lymphoma (DLBCL) in 29% of cases, follicular lymphoma (FL) in 23% of cases and mantle cell lymphoma (MCL) in 5% of cases. CLL patients harboring *PAX5* non‐coding mutations were preferentially associated to the IGHV‐mutated subgroup but were not related to other recurrent CLL mutations, with the exception of del13q14, suggesting that *PAX5* non‐coding mutations are early events in the development of the disease. A second study identified non‐coding *PAX5* enhancer mutations in 3/13 (23%) of CLL cases. In contrast to the first description of *PAX5* mutations, in this study the identified *PAX5* mutations co‐existed with *MYD88, ATM, NOTCH1, SF3B1* and *ZMYM3* mutations (Rose‐Zerilli *et al*., 2016). Finally, a recent study of 46 CLL cases identified *PAX5* non‐coding mutations in 17.4% of CLL cases, which correlated with non‐coding elements of transcription elongation sites, and promoter and enhancer regions. These *PAX5* promoter mutations were found in 22% of IGHV unmutated patients, confirming previous studies (Burns *et al*., 2018).

## 5 Immunoglobulin gene mutations

In normal B lymphocytes, activation‐induced deaminase (AID) is required for the productive generation of antibody diversity by inducing SHM of IGV region and by mediating IGH class‐switch recombination during the development of protective effector mechanisms (Peled *et al*., 2008; Stavnezer *et al*., 2008). The on‐target AID activities consist in the conversion of cytidine to uridine on single‐stranded DNA at the IG locus during germinal center reaction. Functional evidence indicates that BCR pathway activation in CLL derives from contacts between tumor cells and antigens, which are influenced, among other factors, by the SHM action of the rearranged IGHV genes (Vardi *et al*., 2014). The IGHV genes of CLL can accumulate variations as a consequence of the SHM process. The prevalence of mutated IGHV genes is higher among newly diagnosed and asymptomatic CLL patients (~ 60%), whereas the prevalence of IGHV unmutated genes is higher among progressive (~ 50–60%) and relapsed/refractory (~ 70–80%) CLL patients.

Whole‐genome sequencing analysis of a variety of human tumors revealed a new type of off‐target AID activity and related deaminases, revealing multiple mutation clusters of < 10 kb in most of the tumors analyzed (Alexandrov *et al*., 2013; Nik‐Zainal *et al*., 2012). Localized regions of increased mutation density from random substitutions are called kataegis sites and are typically scattered across tumor genomes at a distance of ~ 0.1–1 Mb from each other, revealing 70% multiple mutation cytidine to thymidine transitions clusters. Kataegis mutations occurred in the same DNA strand, by catalytic processivity, and have frequently been associated with genomic rearrangements (Stephens *et al*., 2011). On these bases, kataegis was proposed to be the result of processive cytidine deamination of single‐strand DNA exposed by the resection of double‐strand breaks during DNA repair (Sakofsky *et al*., 2014).

A recent WGS study of 46 CLL patients provided a complete description of non‐coding mutation landscapes of both mutated and unmutated IGHV CLL (Burns *et al*., 2018) (Fig. 1) The study demonstrated that ~ 25% of kataegis non‐coding mutations outside the immunoglobulin loci occurred in genes relevant to CLL. They identified non‐coding mutations in the *ATM* gene that may negatively impact on ATM expression and found non‐coding mutations in the regulatory region of *TCL1A* gene. In particular, analysis of IGHV unmutated CLL cases revealed additional non‐coding mutations in CLL driver genes such as *IKZF3*,* SAMHD1*,* PAX5* and *BIRC3*. Finally, they found that IGHV unmutated CLL harbored coding mutations in driver genes, whereas IGHV‐mutated CLL harbored non‐coding promoter and enhancer mutations caused by aberrant AID activity (Burns *et al*., 2018). Finally, they observed that recurrently non‐coding mutated regions harbored in CLL patients were associated with the concomitant presence of coding mutations, suggesting a functional relevance in CLL pathogenesis.

## 6 Concluding remarks

Current therapeutic approaches involve target proteins; for this reason, non‐coding mutations have only been studied for research purposes and not for medicine cancer care in the clinic. Although non‐coding mutations are related to the protein‐coding gene expressions they regulate, studies of these mutations might help to identify suitable therapeutic approaches to target linked proteins. In CLL, the presence of different genomic lesions demonstrated the enormous biological heterogeneity of this tumor. WGS studies identified non‐coding recurrent mutations, including the 3′‐UTR of *NOTCH1* and a *PAX5* enhancer, resulting in significant activity alterations of these transcription factors genes of well‐known importance in leukemia and other malignancies (Lobry *et al*., 2011; O'Brien *et al*., 2011). Previous studies have shown the effect of *NOTCH1* mutations in CLL prognosis (Puente *et al*., 2011; Villamor *et al*., 2013). However, these studies may seriously underestimate the true incidence of *NOTCH1* deregulation in CLL, considering that ~ 20% of *NOTCH1*‐mutated tumors were also mutated in the 3′‐UTR region. The function of microRNA to regulate gene expression is essential to provide fine control of several cell processes, and deregulation of microRNA may be involved in CLL development/progression. However, more studies are necessary to determine whether microRNA from CLL cells can be used in clinical practice. These independent CLL cohort studies have revealed new driver lesions involved in CLL evolution, helping to clarify the clinical impact of the heterogeneous molecular composition of the disease, resulting in new opportunities for improving the clinical management and personalized treatment of CLL patients.

## Authors contributions

Both authors contributed to the writing of this review article.

## Conflict of interest

The authors have no conflicts of interest to declare.
